# Cytokine-induced senescence in tumors is based on sustained activation of STAT1- and NFκB-dependent gene regulatory signatures

**DOI:** 10.1007/s11357-026-02100-6

**Published:** 2026-01-29

**Authors:** Maximilian Rentschler, Mohamed Ali-Jarboui, Christoph M. Griessinger, Michael Rosen, Grégory Doré, Oliver Bischof, Manfred Kneilling, Martin Röcken, Heidi Braumüller, Thomas Wieder

**Affiliations:** 1 Department of Dermatology, University Medical Center Tübingen, Eberhard Karls University, 72074 Tübingen, Germany; 2 Medical Bioanalytics Institute for Ophthalmic Research, University Medical Center Tübingen, Eberhard Karls University, 72074 Tübingen, Germany; 3 Werner Siemens Imaging Center, Department of Preclinical Imaging and Radiopharmacy, University Medical Center Tübingen, Eberhard Karls University, 72074 Tübingen, Germany; 4 Department of Cell Biology and Infection, Institute Pasteur, Paris , Cedex 15, 75724 France; 5 CNRS Délégation Ile de France Villejuif, 7 Rue Guy Moquet, 94800 Villejuif, France; 6 Cluster of Excellence iFIT (EXC 2180) “Image Guided and Functionally Instructed Tumor Therapies”, 72076 Tübingen, Germany; 7 German Cancer Consortium (DKTK), German Cancer Research Center, Heidelberg, Germany; 8 Department of General and Visceral Surgery, Medical Center, Faculty of Medicine, University of Freiburg, 79106 Freiburg, Germany; 9 Institute of Physiology, Department of Vegetative and Clinical Physiology, Eberhard Karls University Tübingen, 72074 Tübingen, Germany

**Keywords:** Growth arrest, Interferon, Tumor necrosis factor, Immunotherapy, T helper cells

## Abstract

Senescence is a tripartite cellular phenotype characterized by permanent growth arrest, resistance to apoptosis, and high secretory activity. Besides its physiological role in embryonic development and pathophysiological contribution to age-related tissue degeneration, in the context of tumor development senescence is an important suppressor mechanism, that counteracts accelerated proliferation. Among the many stressors that induce senescence is the external stimulation by cytokines. Although cytokine-induced senescence (CIS) has repeatedly been reported in the literature, the signaling networks leading to the senescent phenotype remained enigmatic. Here, we used two models of tumor-associated (TA)-CIS: (i) in vitro treatment of human A204 cancer cells with interferon (IFN)-γ and tumor necrosis factor (TNF) and (ii) in vivo senescence induction by adoptive transfer of T helper 1 (T_H_1) cells into tumor-bearing RIP-Tag2 mice. In both models, gene expression profiling and signal transduction analysis highlighted the persistent activation of IFN-receptor/STAT1 and TNF-receptor/NF-κB/p38 signaling leading to downregulation of cell cycle genes, upregulation of secretory factors and, remarkably, upregulation of pro-apoptotic as well as anti-apoptotic genes. Protein analysis further demonstrated that the growth-arrested tumor cells were not subject to apoptotic death. Altogether, we define a cross-species TA-CIS core gene and signal transduction signature that may be exploited for therapeutic ends for cancer.

**Graphical abstract:**

**Figure:**
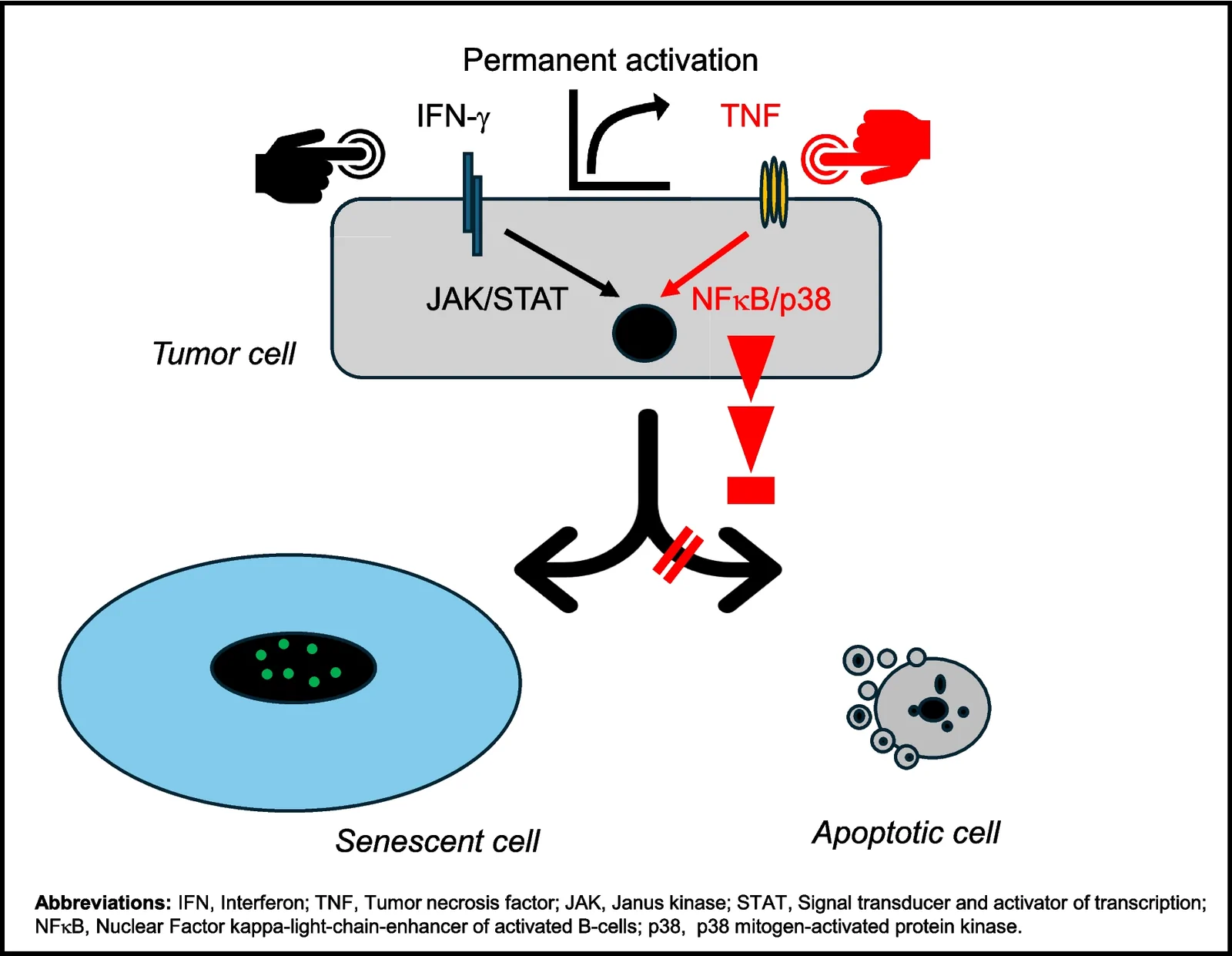

**Supplementary Information:**

The online version contains supplementary material available at https://doi.org/10.1007/s11357-026-02100-6.

## Introduction

Cellular senescence in non-immortalized cells is an intrinsic biological mechanism playing an important role in tissue shaping during developmental processes and in tissue degeneration during aging of organisms (for reviews see [1, 2]). With the detection of senescent cells in human benign naevi of the skin [3] and the description of oncogene-induced senescence (OIS) [4], it became clear that senescence in the context of tumor development (i.e., tumor-associated (TA) senescence) can also function as a barrier against accelerated proliferation and unrestricted growth. On the other hand, as senescent cells persist in the tissue, they can also exert detrimental effects. Accordingly, senescent tumor cell clearance by different immune cells, such as monocytes/macrophages and CD4^+^ T cells, also known as senescence immune surveillance, is imperative for efficient long-term tumor containment [5]. Besides therapy-induced senescence (TIS) [6] and OIS [4], the pro-inflammatory cytokines interferon (IFN)-γ and tumor necrosis factor (TNF) were recently identified as efficient triggers in the context of tumor-associated cytokine-induced senescence (TA-CIS) [7]. However, CIS can occur in different non-immortalized cell types incl. endothelial cells, fibroblasts and melanocytes [8–12] and in cancerous tissues [13–15], and thus, the immune system itself may instrumentalize a senescence response as a biological barrier to control cancer. Congruent with this is the notion that senescence immune surveillance of tumors may be the reason for the long-term survival of melanoma patients during immune checkpoint blockade therapy [16]. According to this concept, patients might benefit from a cancer regimen that turns the deadly disease into a stable one without the necessity of a complete eradication or killing of all tumor cells.

Senescent cells irrespective of their cellular context display a tripartite phenotype, including (i) a persistent proliferative arrest [7], (ii) the secretion of inflammatory cytokines, immune modulators, growth factors, and proteases, a phenomenon called the senescence-associated secretory phenotype (SASP) [17], and (iii) the activation of anti-apoptotic pathways that render the cells apoptosis-resistant [18]. As senescent cells are metabolically active, featuring remarkable protein biosynthesis, glycolysis, and acquisition of a SASP (for review, see [19]), it is likely that the cells activate specific gene regulatory signatures and mRNA expression profiles. In line with this, a transcriptome-based analysis of senescent human papillomavirus-positive cervical cancer cells demonstrated an increased vesicular transport machinery, reflecting the increased secretory activity, and a general downregulation of chromatin modifying factors [20]. A recent study revealed a senescence gene signature of 50 RNAs consistently increased and 18 RNAs consistently decreased across eight different models of senescence, including replicative exhaustion, exposure to ionizing radiation, and expression of oncogenic HRAS^G12V^ [21]. While the above studies mainly focused on the senescence end point, an integrative, time-resolved analysis of transcriptome and epigenome changes in senescence revealed a hierarchically organized, modular, and dynamic transcription factor network orchestrated by the activator protein 1 (AP-1) acting as a pioneer of the senescence enhancer landscape [22]. In contrast to classical senescence of non-immortalized cells which display an irreversible growth arrest, it seems reasonable to define TA-CIS as a persistent growth arrest with eventual reawakening of the proliferation-prone cells, especially when they show mutations in cell cycle regulating genes [23].

**Fig. 1:**
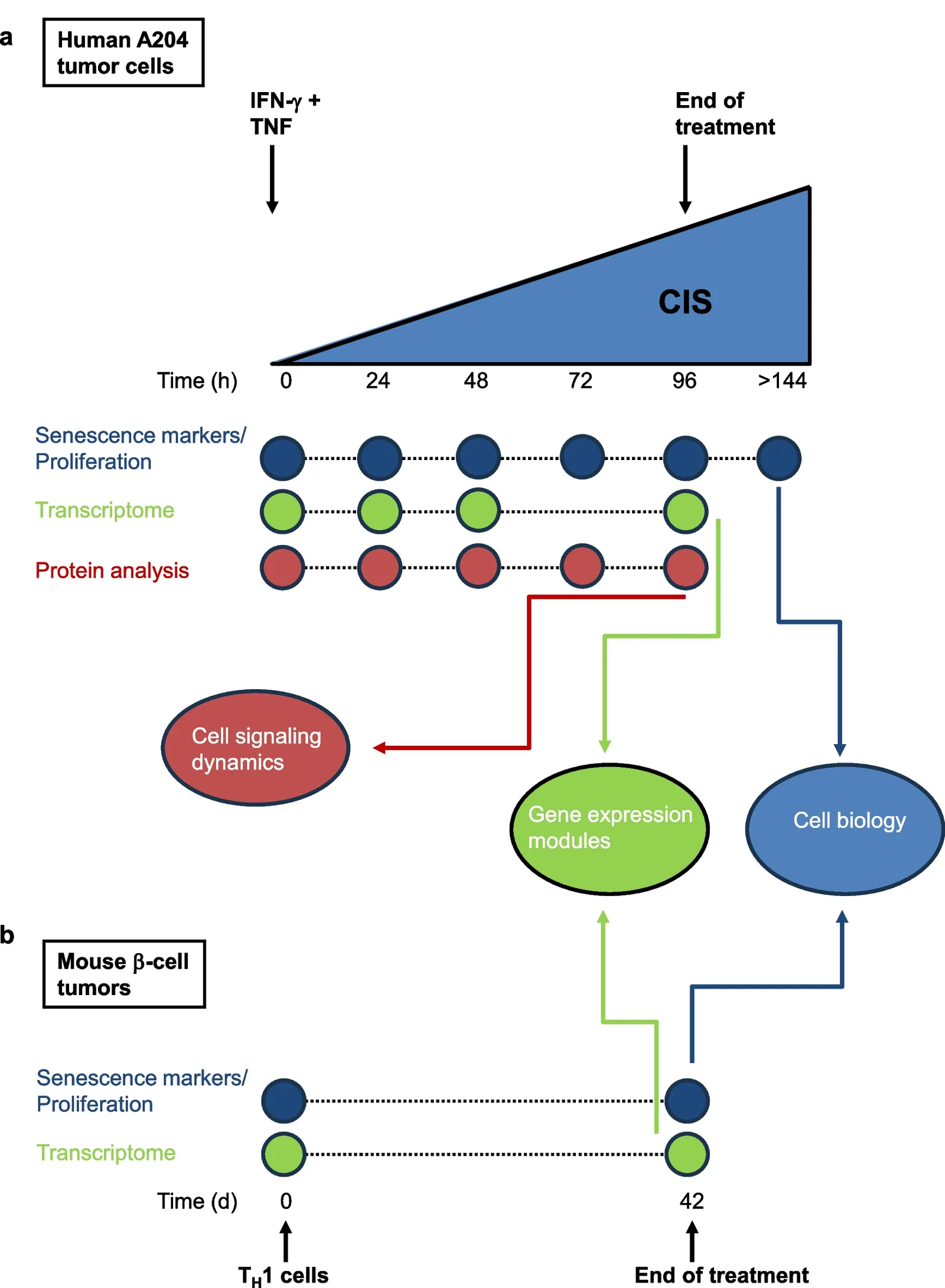
Deciphering the gene-regulatory networks of tumor-associated cytokine-induced senescence. Schematic overview of defining the gene-regulatory networks and protein signaling cascades of tumor-associated cytokine-induced senescence (TA-CIS) in human A204 tumor cells using time-resolved transcriptome (microarray) and protein analysis (Western blot) data sets (**a**) and in mouse β-cell tumors using whole transcriptome (sequencing) data sets (**b**)

In the present work, we used two established TA-CIS models as depicted in Fig. 1: (i) in vitro senescence induction in human A204 rhabdomyosarcoma cells by treatment with IFN-γ and TNF [12], and (ii) in vivo senescence induction in T antigen (Tag)-expressing β-cell tumors of rat insulin promoter (RIP)-Tag2 mice by IFN-γ- and TNF-producing Tag-specific T helper 1 (T_H_1) cells [7, 24]. CIS starts with the concomitant activation of the respective cytokine receptors, i.e., immediately after the addition of IFN-γ and TNF to the culture medium. Thus, by combining time-resolved transcriptome and signal transduction pathway analyses, we could render an in-depth portrayal of TA-CIS dynamics in vitro. Comparative analysis of the in vitro and the in vivo senescence model identified cross-species relevant TA-CIS core factors. Finally, protein analysis of cytokine-treated A204 cells confirmed the transcriptome signatures of TA-CIS and led to a more detailed description of the senescent phenotype of tumor cells.

## Methods

### Animals, induction of in vivo senescence, and isolation of tumors

C3HeB/FeJ (C3H) mice from The Jackson Laboratory, transgenic RIP-Tag2 mice [25], and TCR2 mice [26], all on a C3H background, were bred in-house under specific pathogen-free conditions with free access to food and water ad libitum. Animal experiments were performed by trained research and animal care staff according to animal use and care protocols of the German Animal Protection Law and approved by the local authorities (Regierungspräsidium Tübingen, Germany).

Tag-specific T_H_1 cells were isolated and expanded from female TCR2 mice and characterized by flow cytometry before use in adoptive transfer experiments exactly as described by Griessinger et al. [24]. Senescent tumors were induced in RIP-Tag2 mice by weekly administration of Tag-specific T_H_1 cells for up to 10 weeks [7, 24]. In detail, a 2-Gy whole-body irradiation at a delivery rate of 6 Gy/min was applied to 5-week-old RIP-Tag2 mice using a Gammacell© 1000 Elite unit (137Cs source; Nordion International, Ottawa, Canada), and irradiation was repeated after 6 weeks of therapy. For senescence induction, 10^7^ Tag-T_H_1 cells were injected i.p. in 500 µl 0.9% NaCl into irradiated mice (*n* = 4) once a week. As a control group (Sham, *n* = 4), 500 µl 0.9% NaCl i.p. were injected into irradiated RIP-Tag2 mice at the same time points. After treatment, mice were euthanized and pancreatic tumors were isolated from sham- and from Tag-T_H_1-cell-treated female RIP-Tag2 mice by collagenase digestion (1 mg/ml, Serva) for 10 min at 37 °C. Single tumors were separated under a dissection microscope (Leica Microsystems) and further processed as described below.

### Monitoring of tumor progression

Tag2 inhibits tumor suppressors p53 and retinoblastoma, thus promoting multistage malignant transformation of 20% of the β-cells into insulin-producing insular cell carcinomas [25]. As a result, untreated RIP1-Tag2 mice develop endogenous tumors and die from hypoglycemia at approx. 15 to 17 weeks of age due to the undamped release of insulin by the insular carcinomas. A decrease in blood glucose levels (BGL) therefore unambiguously reflects tumor progression. Consequently, we monitored the BGL as marker of tumor burden (reflecting the tumor size and the number of tumors) twice weekly using a HemoCue Glucose 201 system (Hemocue AB, Ängelholm, Sweden) as established in our laboratory. The blood was freshly drawn from the tail vein and collected in a HemoCue 201 cuvette on the same week days (Monday and Friday) and same time (10 a.m.) to minimize circadian influences on the BGL (for blood glucose levels after start of the Tag-T_H_1 therapy see [24]). In this experimental setting, blood glucose levels below 30 mg/dl were defined as a humane endpoint. None of the animals of the two experimental groups reached this end point or died before meeting the criteria for euthanasia [24].

### Tumor number and typical tumor size in RIP1-Tag2 mice

A blood glucose level of 30 mg/dl is an indirect measure of the tumor mass. Sacrificed mice have typically 8–12 quite small insulin-producing islet cell tumors. The largest islet cell carcinomas have a maximum diameter of approximately 4–5 mm. In our hands, treatment of RIP1-Tag2 mice with Tag-specific T_H_1 cells leads to a significant reduction of the tumor number from 12–16 tumors/pancreas (sham-treated) to 5–6 tumors/pancreas (Tag-T_H_1-cell-treated), and a reduction of the tumor diameter from 1.8–2.4 mm (sham-treated) to 1.0–1.4 mm (Tag-T_H_1-cell-treated) [27].

### Cell culture and in vitro senescence induction by IFN-γ and TNF

Human A204 rhabdomyosarcoma cells [7, 15] were a generous gift by K. Schilbach (University of Tübingen, Germany) and grown in RPMI 1640 medium containing stable l-glutamine and supplemented with 10% fetal calf serum, HEPES buffer (10 mM), sodium pyruvate (1 mM), non-essential amino acids (MEM, at the appropriate concentrations) and penicillin/streptomycin (100 U/ml). All cell culture materials were purchased from Sigma. A204 cells were seeded at a subconfluent density of 1 × 10^4^/cm^2^. Starting the next day, cells were treated for 6–96 h with 100 ng/ml recombinant human IFN-γ (R&D Systems) and 400 pg/ml recombinant human TNF (R&D Systems) to induce permanent growth arrest and TA-CIS [15]. After the indicated incubation time, adherent cells were used for different analyses, as described below.

### Proliferation assay

To characterize the cytokine-induced effects during the treatment phase (6–96 h), the proliferation of A204 cancer cells was measured by [^3^H]-thymidine incorporation essentially as described [15]. Briefly, the cells were seeded at a density of 5 × 10^3^ cells/well onto 96-well flat-bottom plates. After a lag phase of 24 h to allow adherence, the cells were either treated with IFN-γ and TNF in combination or with each cytokine alone, while the controls were incubated with a complete medium containing PBS. [^3^H]-thymidine (0.25 μCi/well) was added 24 h before the respective plates were harvested and finally measured using a FilterMate harvester and a MicroBeta TriLux counter (all reagents and equipment from PerkinElmer), except for the short-time samples that only received [^3^H]-thymidine for 6 h and 12 h, respectively. Data are given as counts per minute (CPM) for each 96-well flat-bottom plate.

### Measurement of growth arrest, senescence-associated β-galactosidase activity, and nuclear staining of p-HP1-γ in A204 cells and tumors

TA-CIS was verified by determination of three established senescence markers: induction of permanent growth arrest, senescence-associated β-galactosidase (SA-β-gal) activity, and nuclear recruitment of phosphorylated heterochromatin protein 1γ (p-HP1γ) into senescence-associated heterochromatin foci (SAHF) [7].

The growth assay was performed by counting living cells after 96 h of cytokine treatment, followed by 1 to 2 subsequent passages without the cytokines as described [7, 15]. Permanent growth arrest is achieved when the cells do not restart exponential growth after the removal of the cytokines.

Briefly, the SA-β-gal activity assay was performed after cytokine treatment using the β-Galactosidase Staining kit (United States Biological), and quantified by counting SA-β-gal^+^ cells using a Zeiss Axiovert 200 microscope (Zeiss) as described [15]. Data are given in %, i.e., SA-β-gal^+^ cells/total cells.

Nuclear staining of p-HP1γ was performed as described earlier [7]. In brief, fresh frozen cryostat sections of β-cell tumors from sham- or Tag-T_H_1-treated RIP-Tag2 mice, or A204 cells grown on chamber slides were stained with rabbit anti-p-HP1γ antibody (1:100; Abcam) in combination with a Cy2-conjugated goat anti-rabbit antibody (1:200, Dianova GmbH). Nuclei were stained with DAPI (Invitrogen, purchased via Thermo Fisher Scientific). The samples were analyzed with an Axiovert 200 microscope from Zeiss equipped with the VisiView software (Visitron Systems).

### RNA extraction and purification after in vitro senescence induction in A204 cells

Total RNA was extracted from A204 cells after treatment with IFN-γ and TNF for 0–96 h essentially as described [15]. In some experiments, A204 cells were incubated for 96 h in a culture medium only (96 h; Ctrl.). Samples were harvested after the indicated time points and total RNA was then isolated using the NucleoSpin RNA kit from Macherey–Nagel according to the manufacturer’s instructions. Afterward, RNA samples were sent on dry ice to the laboratory of O. Bischof at the Institute Pasteur in Paris, France, for further analysis.

### Microarray analysis

Quality control of all RNA samples from A204 cells was performed with the Agilent 2100 Bioanalyzer system and only samples with a RIN score greater than 8 were used for the subsequent analysis. Per sample, 100 ng RNA was then analyzed with Affymetrix Human Transcriptome Arrays 2.0 following the manufacturer’s instructions. After measurement, raw data were provided as CEL files for bioinformatical processing.

### Data analysis

Bioinformatical analysis of the gene expression of A204 cells was performed as follows. CEL files were uploaded and analyzed using the transcriptome Analysis Console (TAC version 4.0, Thermo Fisher Scientific). Default parameters were used for the HTA-2-_0 array against the Human genome hg19 version. Only genes expressed in at least two of the three biological replicates per group were considered. Differentially expressed genes were identified using the Bayes ANOVA method with a gene and exon level *p*-value < 0.05; only protein-coding sequences were used for subsequent analysis. Gene expression of signaling pathways was visualized using pathway maps of the Kyoto Encyclopedia of Genes and Genomes (KEGG) [28]. Copyright permission to use the KEGG pathways “map04630 JAK-STAT signaling pathway, map04668 TNF signaling pathway, map04210 Apoptosis, and map04110 Cell cycle” was granted by Kanehisa Laboratories.

### Western blot analysis

Western blot analysis was performed essentially as described [7, 15]. After cytokine treatment, whole-cell lysates from A204 cells were prepared using RIPA buffer supplemented with a protease and phosphatase inhibitor cocktail (cOmplete and PhosSTOP, respectively; both from Roche Diagnostics), and the protein content per sample was determined with the Pierce BCA Protein Assay kit (Thermo Fisher Scientific). Per sample, 10 µg of protein were separated by SDS-PAGE, transferred onto Immobilon-FL PVDF membranes (Merck Millipore), and blocked 1 h with Intercept Blocking buffer (or its predecessor Odyssey Blocking buffer; both from Li-Cor Biosciences). The membranes were then incubated overnight with a combination of primary antibodies directed against the target of interest and β-actin. For protein analysis, the following antibodies were used: anti-β-actin (1:1000; Cell Signaling Technology, #4970), anti-β-actin (1:5000; Merck Millipore, MAB1501R), anti-phospho-STAT1 (Tyr701) (1:1000; Cell Signaling Technology, #9167), anti-STAT1 (1:1000; Cell Signaling Technology, #9172), anti-phospho-p65 (Ser536) (1:1000; Cell Signaling Technology, #3033), anti-p16 INK4A (1:1000; Cell Signaling Technology, #80,772), anti-p21 Waf1/Cip1 (1:1000; Cell Signaling Technology, #2947), anti-p65 (1:1000; Cell Signaling Technology, #4764), anti-phospho-p38 (Thr180/Tyr182) (1:1000; Cell Signaling Technology, #4511), anti-p38 (1:1000; Cell Signaling Technology, #9212), anti-Caspase-3 (active + pro) (1 µg/ml; OriGene Technologies, AM00162PU-N) and anti-c-IAP2 (1:1000; Cell Signaling Technology, #3130). After washing with TBST, the membranes were incubated 1 h with fluorescent dye-labeled secondary antibodies (either a combination of IRDye 680RD goat anti-mouse IgG (1:15,000) and IRDye 800CW goat anti-rabbit IgG (1:15,000) or IRDye 680RD goat anti-rabbit IgG (1:20,000) and IRDye 800CW goat anti-mouse IgG (1:15,000); all from Li-Cor Biosciences), then washed again and subsequently analyzed with the Odyssey Sa imaging system (Li-Cor Biosciences). Intensity measurements of immunoreactive bands were performed using the Image Studio Life software (Li-Cor Biosciences) and target/reference ratios were then calculated. To analyze additional targets on the same blot, the membrane was stripped with 0.5 M NaOH for 10 min, then washed, blocked, and incubated overnight with the primary antibodies. Afterward, all steps were performed as described above. Cell lysates from HeLa cells were used as a control for most antibodies. Extracts of Jurkat cells treated with cytochrome c (Cell Signaling Technology) were specifically used as a positive control for the cleavage of caspase-3.

### Transcriptome analysis of β-cell tumors

For whole transcriptome analysis, we used tumor tissue from RIP-Tag2 mice, which, after irradiation, received either Tag-T_H_1 cells every week i.p. or sham (500 µl 0.9% NaCl i.p.) treatment for 10 weeks [7, 24]. RNA from formalin-fixed, paraffin-embedded (FFPE) tissue was extracted after macrodissection of RIP-Tag2 tumors from histology slides applying the Maxwell FFPE Tissue LEV RNA Purification Kit (Promega) according to the manufacturer’s protocol. Whole transcriptome sequencing was performed at CeGat GmbH (Tübingen, Germany). One microgram of total RNA per sample was used for library preparation using the Illumina TruSeq Stranded Total RNA Library Prep Kit with rRNA 30 depletion. The sequencing libraries were sequenced on Illumina HiSeq 2500 for a read length of 100 bp in paired-end mode. The quality control of the sequencing resulted in a Q30 value of 94.88%. Unnecessary sequencing reads (pseudogenes, unclassified genes, noncoding regions, etc.) were excluded from the analysis. For bioinformatics analysis, de-multiplexing of the sequencing reads was performed with the illumine bcl2fastq (version 2.19) and was trimmed with Skewer (version 0.1.116). Differential expression analyses between groups were performed with the DESeq2 algorithm [29]. The output reads from the DESeq2 analysis were imported to the Perseus software [30] for statistical data analysis and validation. We filtered the data by applying a t-test with a *p*-value < 0.05 with a minimum of 2 biological replicates required. Experiment reads were separated into 2 groups: Sham and Tag-T_H_1 i.p. Sham values mean was used to calculate the ratios of differential gene expression for each experiment from the Tag-T_H_1 i.p. group. Resulting ratios were normalized, and Log2 was transformed to generate differential gene expression of Tag-T_H_1 i.p. versus Sham. Log2 normalized ratios from each group were submitted to Ingenuity Pathway Analysis (IPA) software (Qiagen). Pathway analysis was conducted on differentially expressed genes with a *t*-test *p*-value cutoff of 0.05 on 15,036 quantified genes. Only genes quantified in the Tag-T_H_1 i.p. group were considered for the pathway analysis. Applying the p-value cutoff of 0.05 resulted in 1963 genes filtered for the Tag-T_H_1 i.p. group.

### Statistical analysis

Data are expressed as arithmetic means ± standard error of the mean (s.e.m). Unpaired Student’s *t*-test was performed when appropriate. An asterisk (*) indicates a *p*-value < 0.05, which was considered statistically significant.

## Results

### Dynamics of cytokine-induced growth arrest and characterization of senescence induction by IFN-γ and TNF in A204 cells

**Fig. 2:**
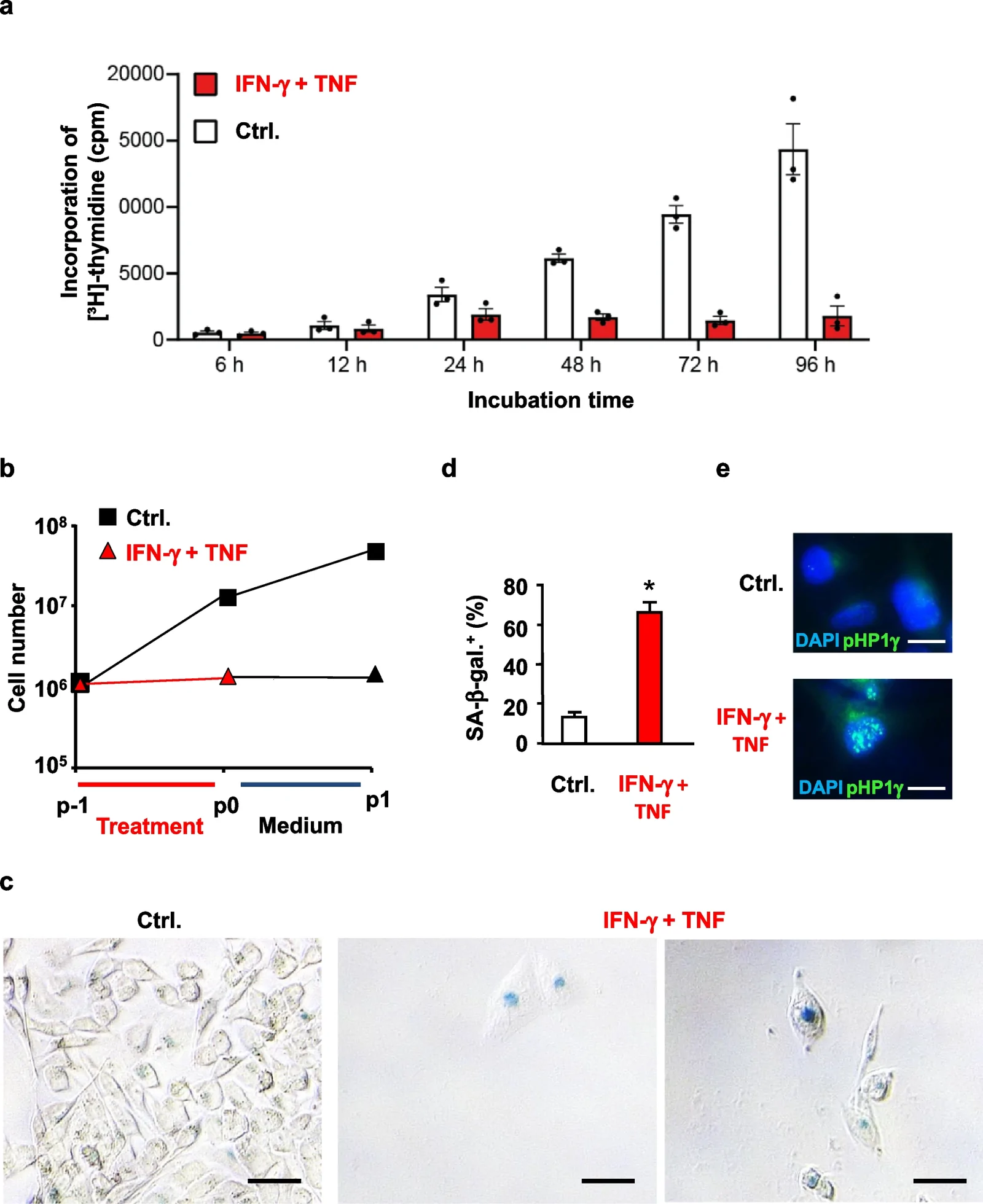
Time course of IFN-γ- and TNF-induced proliferation arrest and characterization of senescent A204 cells. **a** Proliferation of cytokine-treated (red columns) or medium-treated (open columns) A204 cells after different incubation times as measured by [^3^H]-thymidine incorporation (mean ± SD; *n* = 3). **b** Subconfluent A204 cells (passage p-1) were treated for 96 h with medium (Ctrl., black squares) or with medium containing IFN-γ and TNF (red and black triangles). The cytokines were then removed, and the cells were trypsinized, counted, and reseeded at the initial density in medium alone (passage p0). After an additional passage in the absence of the cytokines, the cells were again trypsinized, and the number of viable cells was determined (passage 1). The diagram shows one representative growth arrest out of four. **c** SA-β-gal activity staining of A204 cells after 96 h of medium (left, Ctrl.) or IFN-γ + TNF (right). Bar = 25 µm. **d** Percentage of SA-β-gal^+^ A204 cells treated for 96 h with medium (Ctrl.) or IFN-γ- + TNF (mean ± s.e.m., *n* = 6; *, *p* < 0.05). **e** A204 cells were treated for 96 h with medium (Ctrl.) or IFN-γ and TNF. After incubation, the cells were analyzed by p-HP1γ-staining. Nuclei (blue) are stained with DAPI. Cells with more than 5 nuclear dots are designated as p-HP1γ-positive (7). Representative nuclei of medium (upper, Ctrl.) or IFN-γ- + TNF-treated A204 cells (lower, IFN-γ- + TNF) are shown. Bar, 10 µm

We set up an in vitro model for TA-CIS using the human rhabdomyosarcoma cell line A204 [15]. To precisely demonstrate TA-CIS, we determined five independent markers of senescence in tumor cells: (i) cell cycle arrest by measurement of thymidine incorporation, (ii) growth-arrest which persists for at least one passage after removal of the cytokines, (iii) flattened senescence-like morphology by transmitted-light bright-field microscopy, (iv) induction of senescence-associated-β-galactosidase (SA-β-Gal) activity by the SA-β-Gal assay, and (v) induction of senescence-associated heterochromatin foci (SAHF) by nuclear staining of p-HP1γ. We treated subconfluent A204 cells with an established cocktail of IFN-γ and TNF for 6–96 h. As soon as 24 h after the start of the treatment, IFN-γ and TNF reduced the incorporation of [^3^H]-thymidine into the DNA of the cancer cells. This inhibition of [^3^H]-thymidine incorporation to basal levels remained stable over the whole treatment period of 96 h (Fig. 2a). To confirm the persistent growth arrest of A204 cells, we removed the cytokine cocktail after 96 h. This did not result in a cellular proliferation restart (Fig. 2b).

The diminished incorporation of [^3^H]-thymidine after 96 h of IFN-γ and TNF treatment was reflected in a reduction of the cell density, induction of a flat cytomorphology, a substantial increase in the SA-β-Gal activity of the cells (Fig. 2c, d), and nuclear recruitment of p-HP1γ to SAHF (Fig. 2e), all of which are canonical biomarkers of senescent cells. Taken together, the A204 cell-based in vitro model allows for a detailed dynamic molecular analysis of TA-CIS.

### IFN-γ and TNF activate gene regulatory networks in A204 cells

**Fig. 3:**
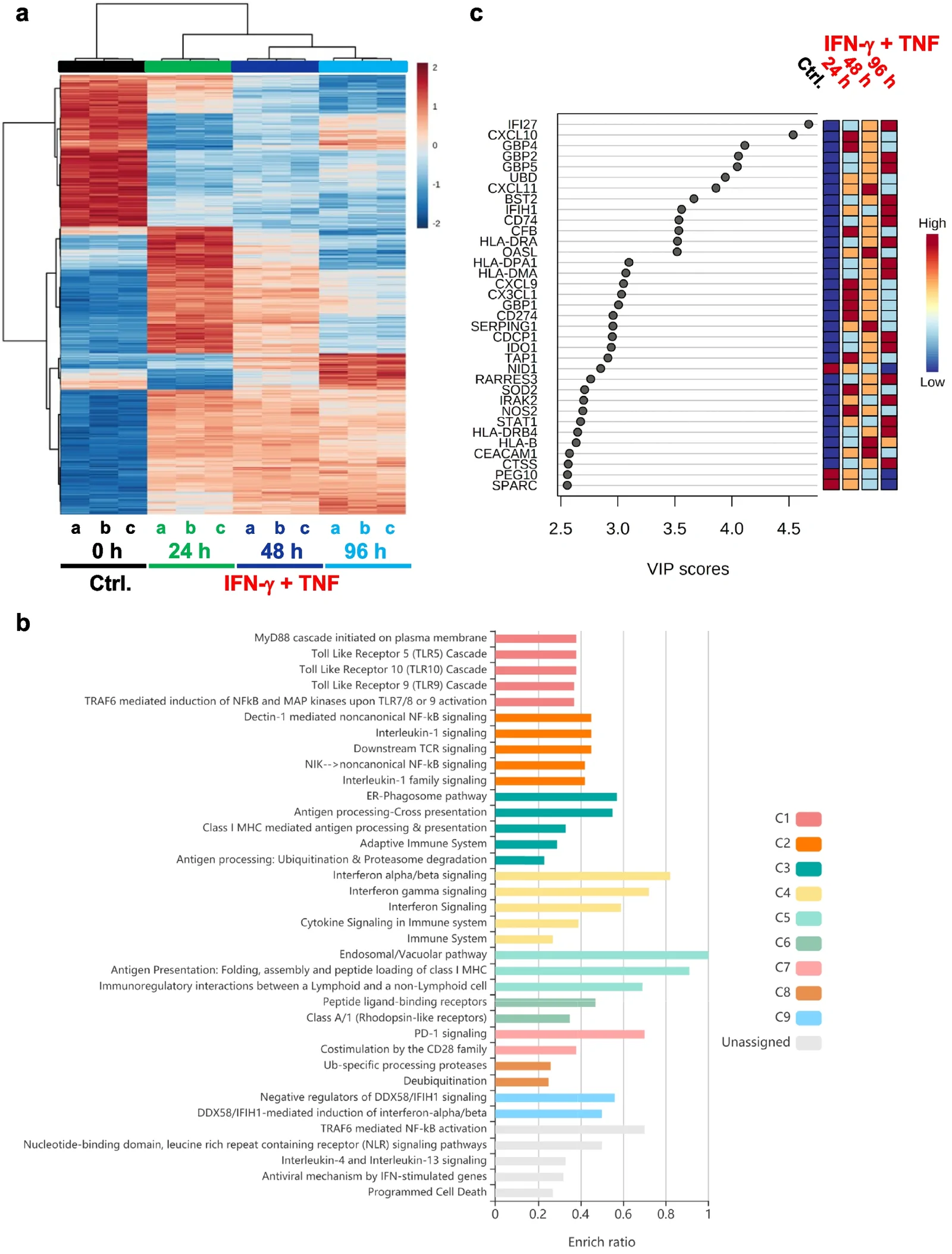
Time course of the IFN-γ- and TNF-induced transcriptional programme in senescence-prone A204 cells. **a** Heatmap showing modules of temporally down- (upper modules) or upregulated genes (lower modules) after treatment of A204 cells with IFN-γ and TNF for 24 h, 48 h and 96 h. Ctrl. = time point 0 h. The Z-score ranging from − 2 to + 2 is shown on the right. (*n* = 3 for all four conditions). **b** Gene ontology enrichment analysis of differentially expressed genes after 96 h treatment of A204 cells with IFN-γ and TNF. The bar chart depicts the enrichment ratios of gene ontology (GO) terms and categories that are assigned to the most tremendously regulated genes identified in A204 cells after 96 h of treatment with IFN-γ and TNF. Different clusters (C1–C9) were formed according to the processes and functions these genes are associated with, except for the GO terms classified as unassigned. **c** The graph shows the VIP score ranking of the top 35 genes that are most relevant for the variance between IFN-γ and TNF-treated cells and the respective medium control (left). The colored boxes on the right side indicate either an increase (red) or a decrease (blue) in the expression of the respective gene by the cytokine treatment at the given time point

To unravel the gene regulatory networks that orchestrate the senescent state in cancer cells, triplicate total RNA samples were extracted from both control tumor cells at time point 0 h and cytokine-treated tumor cells at 24 h, 48 h, and 96 h. After validating the library size distribution quality, the resulting triplicates distinctively clustered on the principal component analysis (PCA) plot (Supporting S1a, b Figure). Transcriptome analyses demonstrated a time-dependent regulation of gene expression following the IFN-γ and TNF treatment, with genes exhibiting dynamic shifts in either upregulation or downregulation compared to time point 0 h. Specifically, genes prominently expressed in proliferating cells experienced a gradual downregulation over time with cytokine treatment, while initially repressed genes were induced. Intriguingly, certain genes displayed an immediate upregulation in response to cytokine stimulation, though their enhanced expression was transient, reverting to baseline levels during prolonged incubation (Fig. 3a). These temporal dynamics in gene expression were further elucidated through Venn diagrams, depicting the unique and shared up- and downregulated genes at each time point. Regarding upregulated genes, a core set of 276 common genes highly responsive to the cytokine cocktail across all time points was identified, whereas 174 genes exhibited consistent repression (Supporting S2a, b Figure).

Further insight into differential gene regulation was gained through a gene ontology (GO) enrichment analysis, focusing on the genes upregulated after 96 h of treatment with IFN-γ and TNF. This analysis revealed color-coded clusters (C1–C9) associated with processes and functions enriched in GO terms. Notably, immune-related processes and functions were significantly upregulated, particularly in clusters C4 (interferon-associated GO terms), C5, and C3 (antigen-related processes). Additionally, multiple processes involved in NF-κB activation were discerned among the GO terms of different clusters (C1, C2) and within unassigned GO terms (Fig. 3b).

Given the cessation of A204 cell proliferation by IFN-γ and TNF, our focus shifted to controlling cell cycle genes. A robust downregulation of most cell cycle genes, including distinct growth factors (e.g., TGF-β) and cyclins/CDKs (e.g., cyclin E/CDK2, cyclin A/CDK1, cyclin B/CDK1), was consistently observed after 96 h of treatment (Supporting S3 Figure). This observation confirmed the profound cell cycle arrest in IFN-γ- and TNF-treated A204 cells. Interestingly, the classical senescence genes CDKN1A and CDKN2A are not regulated on the transcriptional level in A204 cells after treatment with IFN-γ and TNF (Supporting S3 Figure).

Moreover, a Variable Importance in Projection (VIP) analysis was performed to evaluate the relative contribution of different factors to the variance between the time course datasets. The top 35 contributing factors, predominantly representing interferon-regulated genes, underscored the pivotal role of the interferon signature in TA-CIS (Fig. 3c). While most of these genes, including interferon alpha-inducible protein 27 (IFI27) and STAT1, were induced and upregulated in response to challenge with the cytokine cocktail, some exhibited opposing trends (Fig. 3c, right panel). Notably, genes such as NID1, PEG10, and SPARC showed a downregulated expression over time.

**Fig. 4:**
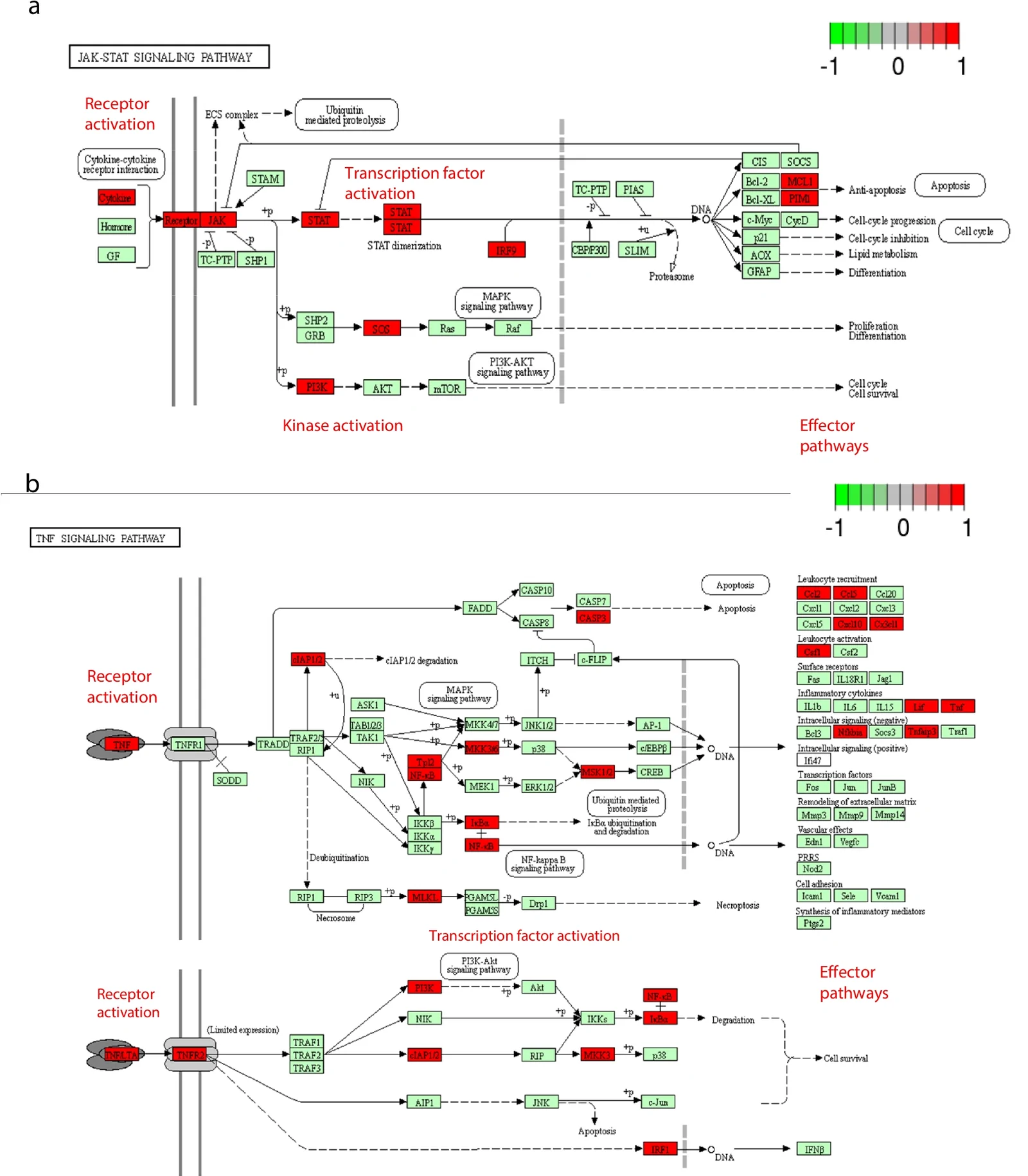
The JAK/STAT signaling pathway and the TNF-triggered stress response are major drivers of TA-CIS in A204 cells. Pathway analysis after 96-h treatment of A204 cells with IFN-γ and TNF identified the IFN-γ-induced JAK/STAT signaling pathway (**a**) and the TNF-driven stress response (**b**) as essential signaling networks leading to TA-CIS. The figures depict parts of the whole pathways with the most tremendously regulated genes. Red, upregulated genes. Green, downregulated genes. CASP3, Caspase-3; cIAP1/2, cellular inhibitor of apoptosis protein-1/2; IRF, Interferon regulatory factor; JAK, Janus kinase; NFκB, nuclear factor “kappa-light-chain-enhancer” of activated B cells; STAT, signal transducer and activator of transcription; TNF, tumor necrosis factor; TNFR, TNF receptor

The subsequent analysis of the essential cytokine-dependent pathways of TA-CIS, namely the JAK/STAT pathway and the TNF pathway (the description of the respective pathways is based on the Kyoto Encyclopedia of Genes and Genomes [28]), revealed profound activation after 96 h. IFN-γ signaling via the JAK/STAT pathway exhibited activation from cytokines to downstream transcription factors and interferon regulatory factors (IRFs), including IRF9. Simultaneously, negative regulators of this pathway, such as suppressors of cytokine signaling (SOCS) and protein inhibitors of activated STATs (PIAS) members, were downregulated after 96 h of cytokine treatment (Fig. 4a). In parallel, a general activation of TNF signaling pathways was observed, commencing with TNF itself, presumably upregulated and induced by an autocrine positive feedback loop. This sustained TNF signaling via TNFR1 and TNFR2 led to prolonged activation of NF-κB and the expression of SASP-related factors associated with downstream effector pathways (Fig. 4b), such as leukocyte recruitment (Ccl2, Ccl5, Cxcl10), leukocyte activation (Colony Stimulating Factor 1 (CSF1)), and inflammatory cytokines (Lif and Tnf).

### IFN-γ and TNF simultaneously stimulate pro- and antiapoptotic genes in A204 cells

TNF is a well-known inducer of apoptotic cell death [31]. Thus, it was unsurprising that the cytokine cocktail led to the upregulation of several proapoptotic genes at all levels of the apoptotic machinery. We found consistent upregulation of death ligands (including TNF itself, see also Fig. 4b) and receptors (TNF Receptor Superfamily Member 1B (TNFRSF1B)), mitochondria-targeting factors (BID), effector caspases (CASP3), and downstream IFN-regulated proapoptotic genes (IRF1, IRF2) (Supporting S4 Figure, yellow circles). According to these data, the IFN-γ and TNF-treated A204 cells should be prone to die. However, the RNA was isolated after 96 h of treatment from adherent A204 cells that were still alive, i.e., senescent. So, we reasoned that the long-term activation of NF-κB (Fig. 4b) might counteract the very strong death-inducing signal. Indeed, in-depth analyses revealed that the cytokine cocktail not only stimulated the proapoptotic pathways but, at the same time, upregulated antiapoptotic factors at several levels of the apoptosis signaling cascade (Supporting S4 Figure, blue circles). IFN-γ and TNF upregulated inhibitory adaptor proteins (CFLAR), negative regulators of the mitochondrial transition pore (MCL1), negative regulators of TP53 (MDM2), and caspase inhibitors (BIRC2, BIRC3). Taken together, the senescent state of cytokine-treated A204 cells is best characterized by a metastable condition that is prone to cell death as soon as the reins are loosened.

### Characterization of the signaling pathways and molecules leading to the senescent phenotype of IFN-γ- and TNF-treated A204 cells

**Fig. 5:**
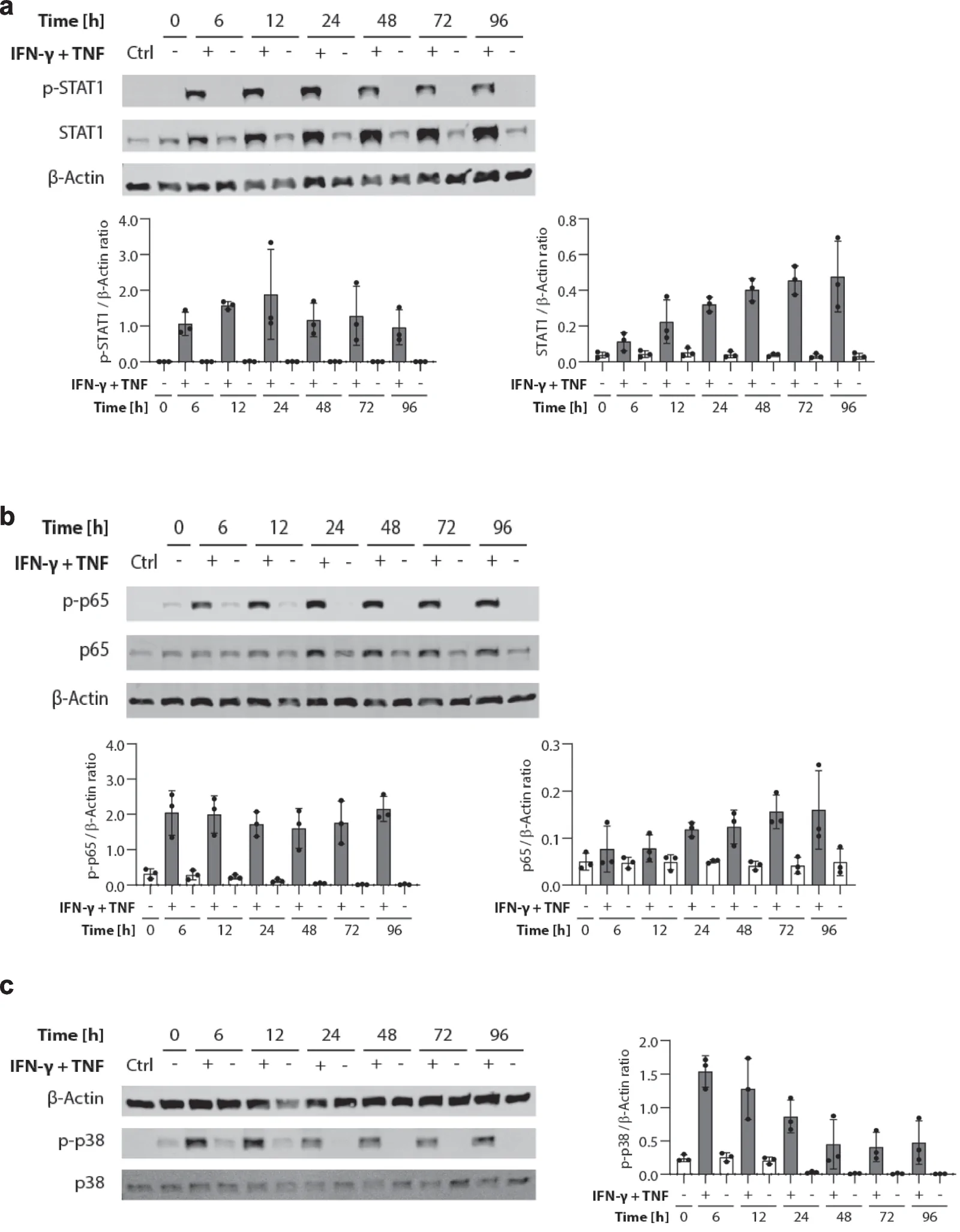
Permanent STAT1 phosphorylation and long-lasting activation of NF-κ-B and p38 signaling during TA-CIS in A204 cells. A204 cells were incubated for up to 96 h in the presence (+) or absence (−) of IFN-γ and TNF. Representative Western blots using specific antibodies detecting p-STAT1 and STAT1 (**a**), p-p65 and p65 (**b**), or p-p38 and p38 (**c**) are shown. HeLa cell lysates were used as a positive control (Ctrl) in all cases, and antibody-staining against β-actin served as loading control. Semiquantative expression ratios in relation to β-actin are shown for p-STAT1 (**a**, left) and STAT1 (**a**, right), p-p65 (**b**, left) and p65 (**b**, right), or p-p38 (**c**) (mean ± SD; *n* = 3)

To confirm the results of the transcriptome analyses, we prepared cell lysates of IFN-γ- and TNF-treated A204 cells. We then determined the crucial players of the unraveled pathways on the protein level using semi-quantitative Western blot analysis. First, we checked the protein expression of two classical senescence markers p16^Ink4A^ and p21, the gene products of CDKN2A and CDKN1A, respectively. The protein analyses revealed that p16^Ink4A^ is not expressed at all in proliferating or growth-arrested A204 cells (not shown). Instead, we observed a fast accumulation of p21 protein in senescent A204 tumor cells as compared with the medium-treated controls in the first 24 h of the treatment phase. Videodensitometric analysis revealed p21 protein accumulation of 313 ± 25% as compared with medium-treated controls (*n* = 3) after 6 h, 343 ± 86% (*n* = 3) after 12 h, and 188 ± 67% (*n* = 3) after 24 h of treatment with IFN-γ and TNF. The p21 protein stayed at this elevated level during the whole 96 h treatment phase, although the cells did not grow to confluency. After 96 h, p21 protein in IFN-γ and TNF-treated cells reached a level of 266 ± 100% (*n* = 3) as compared with the non-confluent control cells at the beginning of the treatment phase. This indicates p16^Ink4A^-independent, p21-dependent TA-CIS in A204 cells as has already been described in human melanoma cells [32]. Next, we checked the IFN-γ signaling cascade and found early phosphorylation of STAT1 after 6 h. Interestingly, STAT1 was constantly activated until the end of the treatment phase of 96 h, and this hyperphosphorylation of STAT1 was further accompanied by the accumulation of the STAT1 protein (Fig. 5a). However, this strong and long-lasting activation of STAT1 was also seen in the single treatment regimen with IFN-γ alone, a condition where the A204 cells already restarted to grow (Supporting S5a, b Figure).

Next, we analyzed the activation of NF-κB. The p65 subunit was also phosphorylated as early as 6 h after stimulation with IFN-γ and TNF. Most intriguingly, the strong phosphorylation of p65 was maintained at the same level until the end of the treatment phase after 96 h. This long-term activation by phosphorylation was made possible by continuous upregulation of the p65 protein itself (Fig. 5b). The activation of the NF-κB pathway was accompanied by stimulation of the stress-associated mitogen-activated protein kinase (MAPK) p38. The protein kinase p38 also showed sustained phosphorylation starting after 6 h and lasting for 96 h (Fig. 5c). This long-term activation of the NF-κB/p38 axis is unique, as the activation of phosphorylation cascades is normally reversible, and the stimulation is stopped either by dephosphorylation or cleavage of the proteins. We therefore checked whether single treatments with the cytokines elicit similar cellular reactions. Neither IFN-γ nor TNF alone was able to induce a state of permanent growth arrest in A204 cells, as the cells restarted growing immediately after the removal of the respective cytokine (Supporting S5a Figure). Further analyses revealed that the single treatments did not induce long-term activation of the NF-κB/p38 axis for up to 96 h but showed reversible phosphorylation that ceased after 24–48 h of TNF treatment (Supporting S5c, d Figure).

**Fig. 6:**
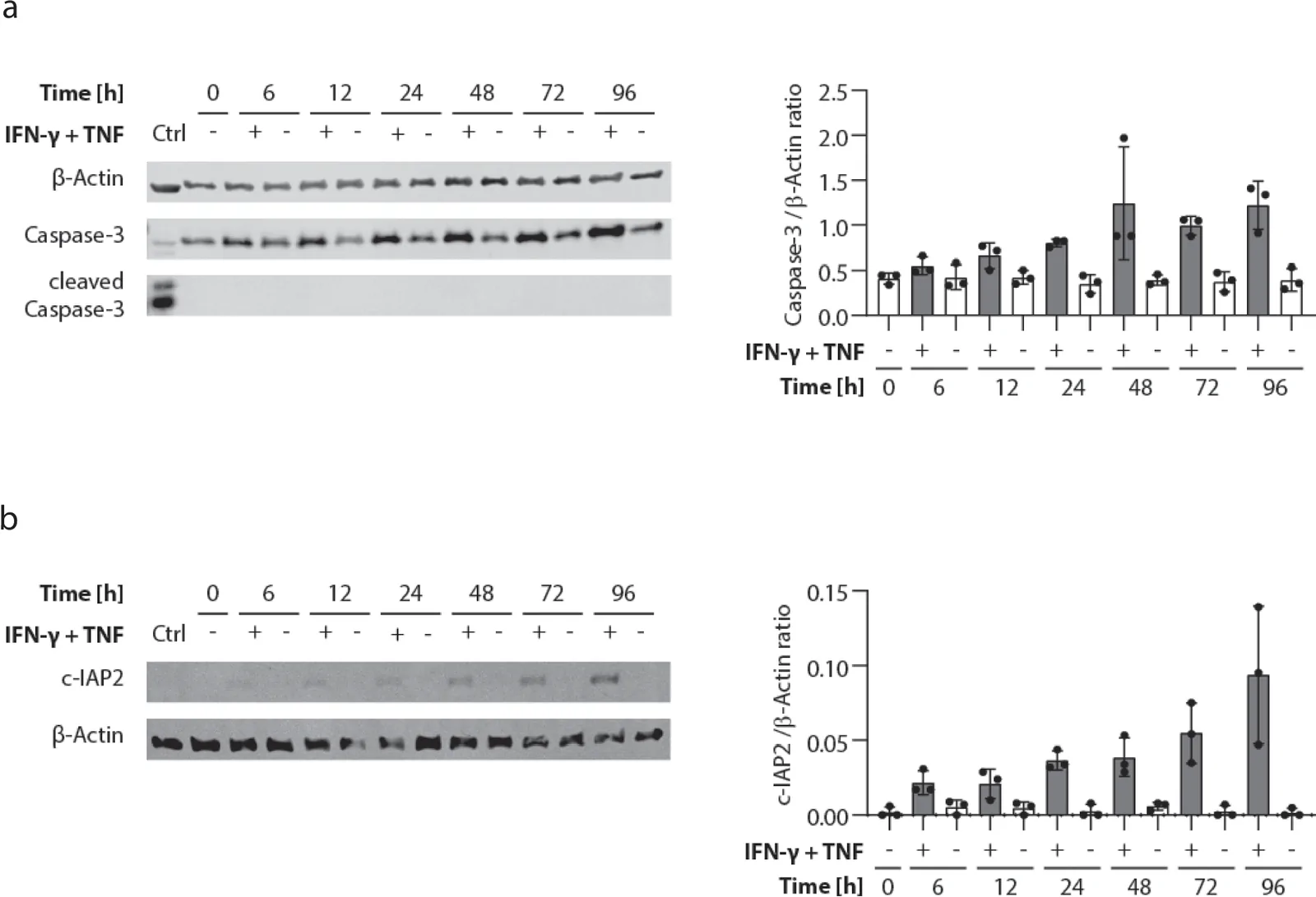
Induction of pro- and anti-apoptotic factors in A204 cells undergoing TA-CIS. A204 cells were incubated for up to 96 h in the presence (+) or absence (−) of IFN-γ and TNF. Representative Western blots using specific antibodies detecting caspase-3 and cleaved caspase-3 (**a**), or c-IAP2 (**b**) are shown. Cytochrome c-treated Jurkat cell lysates were used as a positive control for cleaved caspase-3 (Ctrl), and antibody-staining against β-actin served as loading control. Semiquantative expression ratios in relation to β-actin are shown for caspase-3 (**a**) or c-IAP2 (**b**) on the right (mean ± SD; *n* = 3)

Furthermore, we also investigated the core of the apoptosis cascade on the protein level, i.e., the main executioner enzyme, caspase 3. In accordance with the transcriptome analysis, the levels of the inactive procaspase-3 were increased in IFN-γ- and TNF-treated A204 cells as compared with the medium-treated controls (Fig. 6a, middle lane). In contrast to the included positive control, however, the cells did not contain active, cleaved caspase-3 (Fig. 6a, lower lane), which explains the accumulation of procaspase-3 at the end of the incubation time (Fig. 6a, right). The antiapoptotic phenotype of senescent A204 cells was further elucidated by the determination of the caspase inhibitor c-IAP2. This effective apoptosis inhibitor was steadily upregulated during the course of senescence induction, thereby reaching very high expression levels after 96 h of IFN-γ and TNF treatment (Fig. 6b). Interestingly, neither IFN-γ nor TNF alone induced detectable amounts of antiapoptotic c-IAP2 over time (Supporting S5e Figure). Taken together, analyzing the signaling proteins and pathways of IFN-γ and TNF-treated A204 cells on the protein level confirmed the core gene networks described in the previous chapters, i.e., sustained upregulation of STAT1-dependent IFN-γ signaling, permanent activation of the TNF-driven NF-κB/p38 axis, and consolidation of an effectively halted “preapoptotic” state.

### Core signaling axes of the transcriptomic landscape in Tag-T_H_1 cell-treated β-tumors

**Fig. 7:**
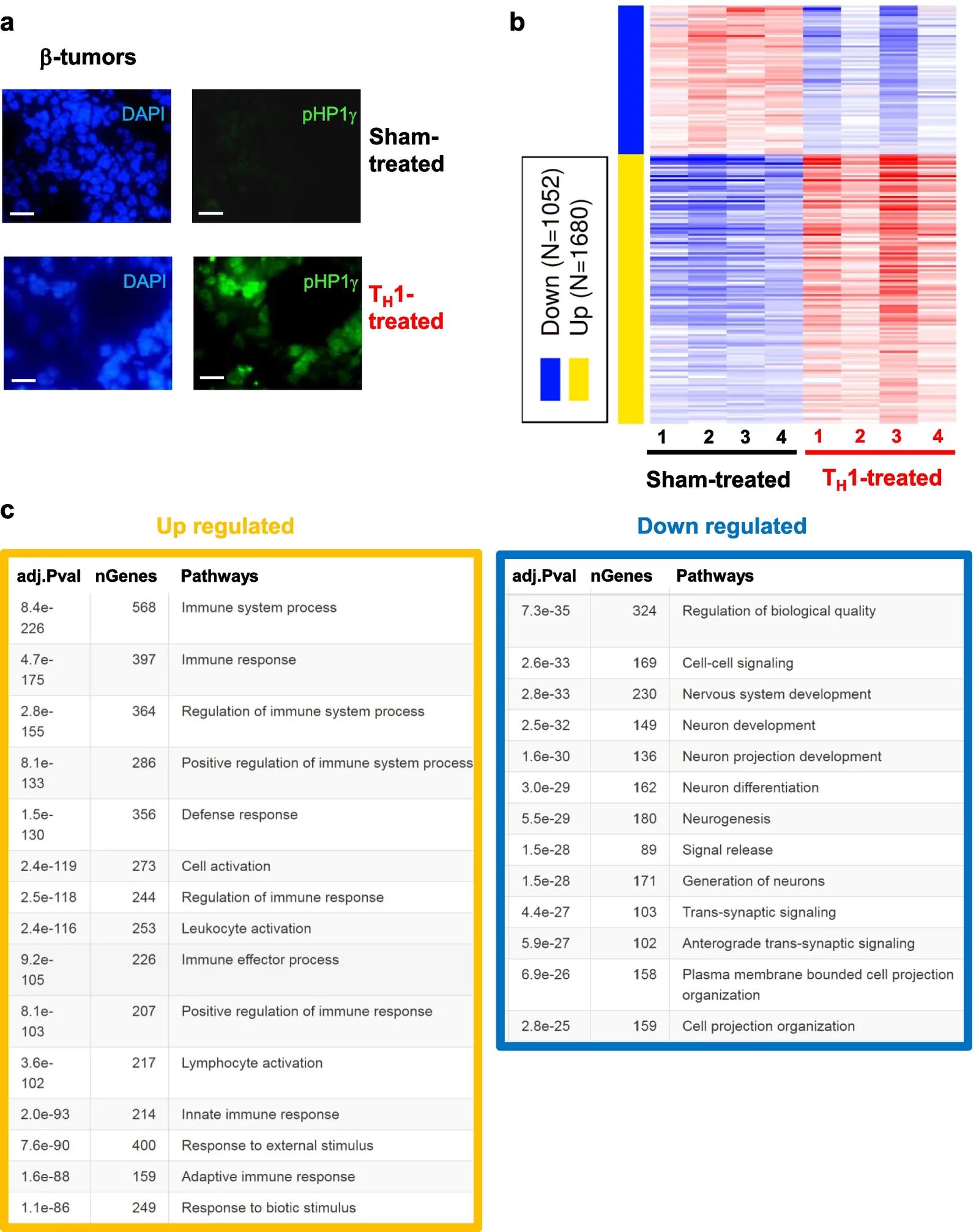
T_H_1-cell-mediated immune therapy activates a distinct transcriptional programme in β-cancer cells. **a** RIP-Tag2 mice were either subject to treatment with tumor-specific T_H_1-cells or a sham treatment. Tumor slices were analyzed by p-HP1γ-staining. Nuclei are blue and p-HP1γ-positive nuclei are green. Bar, 10 µm. **b** Heatmap showing modules of temporally down- (upper module, blue bar on the left) or upregulated genes (lower module, yellow bar on the left) of four replicates of tumors from sham- or T_H_1-treated mice. **c** Functional over-representation of genes in T_H_1-treated tumors associated with different pathways in the upregulated (left, yellow square) or downregulated (right, blue square) module. The number of genes found for each pathway is given in the middle column (nGenes)

To further determine the essential gene networks of TA-CIS in vivo, we analyzed an established model of IFN-γ and TNF-dependent senescence in cancer-prone RIP-Tag2 mice [7, 24]. Starting at week 6 after birth, repeated treatment of RIP-Tag2 mice with Tag-T_H_1 cells i.p. leads to inhibition of tumor development and increased survival of the diseased mice [27]. Using this reliable protocol [7, 24], RIP-Tag2 mice were treated with Tag-T_H_1 cells i.p. or sham. Afterward, whole β-tumors were isolated from the pancreas of the mice. To prove senescence induction in the β-tumors, we stained tumor samples from sham- and Tag-T_H_1-treated mice for the senescence marker p-HP1γ. As expected, β-tumors from sham-treated mice were virtually free of p-HP1γ staining, whereas β-tumors from Tag-T_H_1 cell-treated mice showed strong induction of the p-HP1γ staining (Fig. 7a). These data confirmed that the β-tumors were driven into senescence (for details and analysis of other senescence markers, see also reference [24]), and exhibited senescence-associated chromatin changes.

Next, we compared the transcriptome of proliferating and growth-arrested β-tumors in RIP-Tag2 mice. For this, we isolated RNA from sham- and Tag-T_H_1 cell-treated tumors (for quality control, see Supporting S6 Figure).

Initial transcriptome analyses revealed differential gene expression in β-tumors of mice treated with Tag-T_H_1 cells (Fig. 7b). By analyzing four replicates of Tag-T_H_1 cell-treated tumors, we found 1052 commonly downregulated genes and 1680 commonly upregulated genes as compared with the four sham-treated controls (Fig. 7b). Gene ontology enrichment analysis revealed that especially the upregulated genes were similar to those of the cytokine-treated A204 cells. Consistent with the results originating from the transcriptome analysis of the A204 cell line experiments in vitro, the upregulated pathways were mainly involved in the immune system process, the immune response, and cell activation (Fig. 7c).

As T_H_1 cells stop the growth of β-tumors in mice [27], we first focussed on controlling cell cycle genes. As in the case of the cytokine-treated A204 cells, we commonly found in the isolated β-tumors a strong downregulation of cell cycle genes, mainly at the level of cyclins and CDKs, e.g., cyclin E, cyclin A/CDK1 and cyclin B/CDK1 (Supporting S7 Figure). Thus, T_H_1-treated mice contain β-tumor cells, which are persistently cell cycle-arrested. As in the case of A204 cells, the classical senescence genes p21 Cip1 and p16 Ink4A are not regulated on the transcriptional level after treatment of the mice with T_H_1-cells (Supporting S7 Figure). Nevertheless, it has been repeatedly shown that T_H_1-cells drive β-tumor cells into p16^Ink4A^-dependent TA-CIS with an upregulation of the p16^Ink4A^ protein [7, 23, 24].

**Fig. 8:**
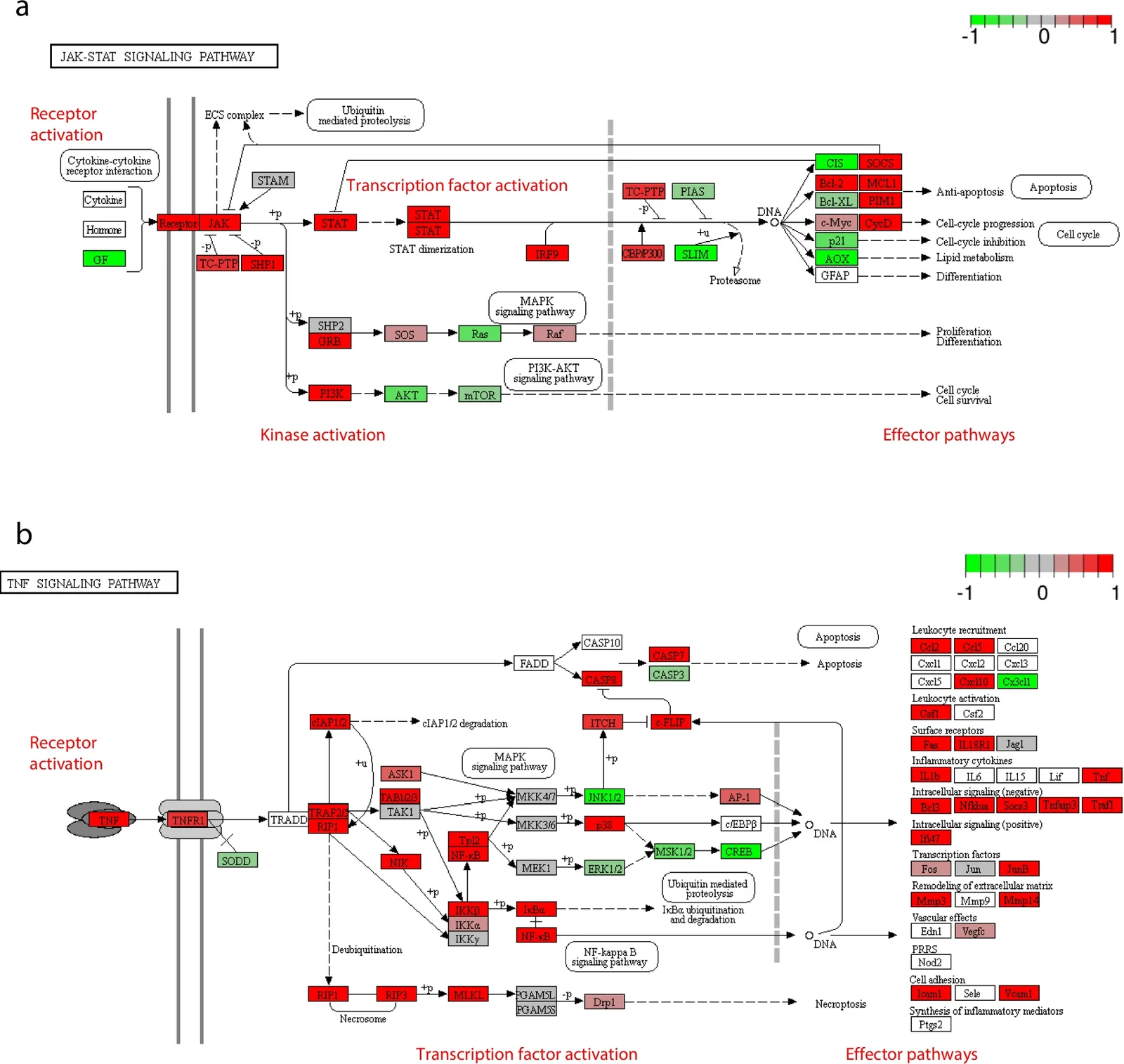
IFN-γ-mediated JAK/STAT signaling and TNF-NFκB-driven stress response in senescent β-tumors after T_H_1 immune therapy. Schemes depicting the relevant genes of the JAK/STAT signaling pathway (**a**) or the TNF-driven stress response (**b**), incl. activation of receptors, transcription factors, and downstream effector pathways. Upregulated genes in tumors of T_H_1-treated mice are marked in red, and downregulated genes are marked in green. The Z-score ranging from −1 to + 1 is shown on the top of each pathway. CASP7/8, caspase-7/−8; c-FLIP, cellular FLICE (FADD-like IL-1β-converting enzyme)-inhibitory protein; cIAP1/2, cellular inhibitor of apoptosis protein-1/2; IRF9, interferon regulatory factor 9; JAK, Janus kinase; NFκB, nuclear factor “kappa-light-chain-enhancer” of activated B cells; STAT, signal transducer and activator of transcription; TNF, tumor necrosis factor; TNFR1, TNF receptor 1

We then focused on the two relevant signaling pathways of CIS, the IFN-γ-STAT pathway and the TNF pathway (the description of the respective pathways in mice is also based on the Kyoto Encyclopedia of Genes and Genomes [28]). We again found profound activation of the IFN-γ-STAT pathway, also at the level of the appropriate receptors and the coupled kinases (JAK, PI3K), leading to recruitment and upregulation of the STAT transcription factors and further activation of different effector pathways, e.g. anti-apoptosis (Fig. 8a). Importantly, the TNF signaling pathway was activated in parallel, starting with TNF itself and its receptor TNFR1, and leading to sustained NF-κB/p38 upregulation and further activation of downstream effector pathways, such as leukocyte recruitment (Ccl2, Ccl5), leukocyte activation (Csf1), and transcription factors (JunB) (Fig. 8b). Taken together, our data clearly demonstrate that the simultaneous and long-lasting activation of the JAK/STAT and NF-κB/p38 signaling axes constitutes the core signaling module of TA-CIS in vitro and in vivo.

## Discussion

Senescence has long been considered as an irreversible and static condition. However, and especially in the context of cancer development, it is now clear that senescence represents a dynamic cellular process that involves cell-autonomous and non-cell-autonomous properties [33]. In the present study, we are able to define tumor-associated cytokine-induced senescence (TA-CIS) as an anti-tumor mechanism that is different from classical senescence in non-immortalized cells with an irreversible growth arrest [1, 2] as described in melanocytes, endothelial cells, or fibroblasts [8–12]. In our hands, TA-CIS shows clear signs of a reduction in cyclins which is consistent with growth arrest. This reduction of cyclins, however, may be reversible indicating that the tumor cells may eventually reawake from persistent growth arrest [23]. Likewise, a transient drug resistance mechanism has recently been demonstrated in an elegant study for therapy-induced senescence in breast cancer cells [34].

Senescent cells show senescence-associated heterochromatin foci (SAHF) with a reduction of the transcription of distinct gene clusters, mainly protein-coding transcripts of cell cycle genes or factors regulating cellular proliferation [15, 35], or histone clusters [21]. In our hands, we found SAHF formation after cytokine-dependent senescence induction in A204 cells in vitro, which coincided with a strong downregulation of the main cell cycle regulator pathways. This might indeed explain the long-lasting effect of IFN-γ and TNF that clearly exceeds the limits of the treatment phase. To identify robust shared signaling networks leading to senescence after cytokine treatment, we have performed time-dependent transcriptome analyses across the whole time grid of senescence induction in A204 cancer cells. The intersection of the altered transcriptomes revealed 276 RNAs consistently elevated and 174 RNAs consistently reduced at all time points. Most strikingly, we found an IFN signature with profound upregulation of the JAK/STAT signaling axis, a phenomenon that has also been described in drug-induced cellular senescence [36]. In their study, the authors showed expression of JAK/STAT-activating ligands, such as IFN-γ, IFN-β and IL-6, by senescent cells, supporting the idea of a self-sustaining autocrine or paracrine activation of the JAK/STAT axis. To test this in our system, we analyzed the activation status of STAT1 by Western blot using phospho-specific antibodies. STAT1 is activated through phosphorylation at tyrosine residue 701, a site known to mediate STAT1 dimerization and to be phosphorylated by different kinases and JAK family members [37–39]. Not astoundingly, we found phosphorylation of STAT1 immediately after receptor activation in the first 6 h of treatment. However, the activation persisted over the whole incubation period of 96 h. This might thus explain the predominant role of the interferon signature in the process of CIS induction. In addition, it stands for the interferon-dominated effects found in the transcriptomic signature after 96 h, with CXCL10 being the second most upregulated gene. STAT1 has previously been described to be a shared upregulated RNA in eight diverse models of senescence, incl. replicative exhaustion, exposure to ionizing radiation or doxorubicin, and expression of the oncogene HRAS^G12V^ [21], and its functional role in CIS has been demonstrated [7, 23]. The importance of the IFN signature was likewise shown by comparing the transcriptome of senescent MCF-7 cells after irradiation with the effects of an induced overexpression of p21 [40]. Wu et al. discovered a core network of shared up- and downregulated genes in both conditions. Although they found differences between the two groups, they identified IFNGR1, STAT1, IRF1, or IRF9 to be upregulated in the irradiated MCF-7 cells. The critical role of the STAT/IRF1 axis during cytokine release syndrome-associated senescence was also demonstrated in alveolar epithelial cells [41]. Thus, an overwhelming bulk of data suggests that STAT1 is one of the main players in different senescence signaling networks. Possibly, this is a reminiscence of the original function of IFN pathways in defending the cell against viral infections by stopping cellular replication and proliferation.

Since the JAK/STAT axis is also activated in response to type I interferons, e.g., IFN-α and IFN-β, the signal transduction of CIS may not be limited to STAT1. While an IFN-γ response is transduced via STAT1 homodimers, the other interferons use additional STAT family members for their signaling which leads to the formation of different STAT (hetero-)dimers. As *STAT2* and *STAT3* were also found to be upregulated after challenge with IFN-γ and TNF, the applied combination of cytokines probably leads to convergent signaling events or to the secretion of other factors that might explain their transcription. Interestingly, previous reports found that STAT3 contributes to senescence in different systems [42, 43]. Our results together with the data of other groups therefore strongly indicate that STAT family members comprise an important module of the core network leading to CIS.

Aside from JAK/STAT, TNFR1-related signaling is a necessary driver of TA-CIS [7, 27]. The TNF pathway engages various factors including the stress-activated MAPK p38, which is also involved in the non-canonical signaling of different interferons [44], and its activity has already been associated with stress-induced senescence [45, 46]. We thus analyzed p38 in the context of CIS. In contrast to STAT1 and other highly induced factors related to IFN-γ signaling, none of the p38 family members was upregulated on the transcriptional and protein level during the treatment phase. On the other hand, the phosphorylation and thus activation pattern was similar to that observed for STAT1. The treatment with both cytokines induced a persistent activation of p38 for up to 96 h as seen by the phosphorylation of threonine 180 and tyrosine 182. In clear contrast, the single treatment with TNF mediated only transient effects on p38 phosphorylation during the first 24 h. Similar to the activation of p38 MAPK signaling, another important factor that contributed to the process of senescence induction was NF-κB. The transcriptome analysis showed upregulation of relevant genes associated with NF-κB signaling (i.e., *RELA*, *NFKB1*, *NFKB2*, *NFKBIA*, and *TNFAIP3*), and the expression of the subunits p65 (encoded by the *RELA* gene) was further analyzed on the protein level. Similar to STAT1 and the p38 MAPK, a strong phosphorylation of NF-κB p65 was present during the whole treatment phase with IFN-γ and TNF that was accompanied by the accumulation of the total p65 protein. In contrast, the initial phosphorylation induced by single treatment with TNF was rapidly abolished after 24 h, whereas treatment with IFN-γ alone completely failed to phosphorylate the p65 subunit of NF-κB at all. A similar phosphorylation pattern of NF-κB p105 was present that again only prevailed in the treatment schedule with both cytokines and quickly faded after stimulation with TNF alone. In this case, phosphorylation of the serine residue 933 and at other sites of the precursor molecule is mediated by the IκB kinase (IKK) complex and causes the subsequent processing of p105 via the ubiquitin proteasome system [47]. Similarly, the observed phosphorylation of p65 at Ser536 is facilitated by different kinases including the IKKs and has been generally associated with the activation of NF-κB after stimulation with different factors such as TNF or lymphotoxin [48, 49]. A study based on a phosphomimetic p65 mutant linked phosphorylated Ser536 to the induction of apoptosis and senescence [50], whereas inhibition of the NF-κB/p38 axis by colchicine inhibited tobacco smoke condensate-induced senescence in endothelial cells [51]. In addition, a novel biphasic pattern of NF-κB activation was currently identified in senescent cells [52]. Thus, our data that TA-CIS needs persistent activation of the TNF/NF-κB/p38 axis likewise point into the direction that senescent cells are kept in a state of halted apoptosis. This shall render those cells particularly sensitive for final destruction. Regarding the cytokines used in the present study, cell protective effects of NF-κB have also been described by others [53, 54]. In this line, Thapa et al. identified the manganese-dependent superoxide dismutase MnSOD (encoded by sod2) as another important factor that counteracts accumulation of ROS and prevents cell death induction through IFN-γ [55]. This finding might translate to TA-CIS, as upregulation of the antioxidant gene SOD2 was also observed in A204 cells after cytokine treatment with IFN-γ and TNF.

Senescent cells are very robust, long-living cells which accumulate in tissues. Thus, approximately 20 years ago two breakthrough articles pushed forward senescence research by demonstrating the presence of senescent cells in precancerous lesions or tissues in vivo [3, 4]. In line with this, we could demonstrate the induction of cancer cell senescence by T_H_1-cell-mediated immunity in β-cell tumors [7, 23]. In the present study, we therefore used this in vivo model of TA-CIS to validate the core gene and signal transduction signature of the A204 cells. Indeed, we showed that similar signatures and pathways, namely downregulation of cell cycle genes, simultaneous activation of the IFN-γ-STAT axis and upregulation of the TNF pathway with downstream activation of NF-κB/p38, were present in the senescent tumors. Interestingly, we could also find the upregulation of distinct downstream target genes, e.g., VCAM1, CCL2, and Jun, which have recently been described to be highly expressed in senescent human peritoneal metastases in vivo [56].

In conclusion, our data broaden the understanding of the underlying cellular events and signaling networks that finally lead to the senescent state of TA-CIS. By combining computational modeling to analyze complex biological data sets (i.e., the global transcriptome of cytokine-treated cells and its modulation over time as compared with the global transcriptome of T_H_1-immunity-induced dormant tumors) and functional analyses to determine the cellular outcome (i.e., protein analyses of highly regulated targets in terms of general expression and their activation), we were able to identify the fundamental STAT- and NF-κB-dependent networks of gene regulation as well as the cellular signaling cascades driving tumors into senescence. As a result of the continuous treatment with pro-inflammatory cytokines, a hyperactivation of the respective signaling pathways was triggered that only in conjunction mediated the cytostatic effects. Through the accumulation of non-lethal stress during the 96 h of treatment, TA-CIS phenotypically resembles a proapoptotic state with a distinct SASP as recently demonstrated by Homann et al. [32] where apoptosis is indeed induced but not executed. Our findings are in line with the original observation that TA-CIS mainly occurred in cells escaping from IFN-γ- and TNF-mediated apoptosis [7, 27] thereby shaping the tumor microenvironment by cytokine-mediated effects on different cell types incl. immune cells, endothelial cells, fibroblasts, macrophages etc. (for reviews, see references [57, 58]). Interestingly, persister and senescent cancer cells are distinct states of cell cycle arrest that differ in their inflammatory gene signatures with persister cells having lost their capacity to produce a SASP [59]. At the same time, however, the phenomenon that senescent cells are resistant to apoptosis is also their Achilles’ heel that renders these growth-arrested cancer cells vulnerable for the subsequent application of senolytic drugs. These substances target members of anti-apoptotic pathways and lead to the selective elimination of senescent cells [60], which is a quite novel and promising strategy in cancer treatment [61].

## Supplementary Information

Below is the link to the electronic supplementary material.

ESM 1 (PPTX 1.53 MB)

## Data Availability

The data underlying this article with the exception of the original files of RNA analyses (e.g. CEL files, Illumina-type sequencing data) are available in the article and in its online supporting information. CEL files and Illumina-type sequencing data are available from the corresponding author and will be deposited after acceptance of the manuscript.
